# Elafin: a double agent in breast and ovarian cancer

**DOI:** 10.18632/oncoscience.246

**Published:** 2015-09-17

**Authors:** Yi Feng, Kai Doberstein, Ronny Drapkin

**Affiliations:** Ovarian Cancer Research Center, Department of Obstetrics and Gynecology, Basser Center for BRCA, University of Pennsylvania Perelman School of Medicine, Philadelphia, PA, USA

**Keywords:** Elafin, high-grade serous ovarian carcinoma, basal-like breast cancer, WAP

Breast and ovarian cancers account for approximately 55,000 deaths each year in the United States [1]. Both tumor types exhibit morphologic and molecular heterogeneity; each with multiple histologic subtypes that differ significantly in their prognosis and response to therapy. The recent characterization of breast and ovarian cancers by The Cancer Genome Atlas (TCGA) revealed that high-grade serous ovarian carcinomas (HGSOC) and basal-like breast cancers (BLBC) share many more genetic features than previously appreciated [2–3]. In particular, both tumor types exhibit a tendency for widespread chromosomal copy number alterations, almost ubiquitous *TP53* mutation, frequent disruption of the *BRCA* pathway, *MYC* gain, *CCNE1* amplification together with *RB* loss, and FOXM1 hyperactivation [3]. These similarities strongly suggested the existence of a common shared molecular pathogenesis.

Both tumor types also share a poor 5-year survival rate in part because there are currently no means available, including serum biomarkers, for the early detection of either HGSOCs or BLBCs. The Whey Acidic Protein (WAP) gene cluster, located on chromosome 20q12-13.2 [4], is frequently amplified in HGSOC [2] and BLBC [3]. This cluster contains 14 genes, each encoding at least one WAP domain. Among these WAP-containing genes is *WFDC2*, the gene that encodes for HE4. We previously showed that HE4 is expressed and secreted by HGSOCs as a glycoprotein [4]. It was subsequently shown to circulate in the bloodstream of ovarian cancer patients and to be more specific for cancer detection than CA125; as such, HE4 is the second FDA-approved biomarker for ovarian cancer (CA125 being the first). Both HE4 and CA125 are used in the clinical setting to monitor patients with ovarian cancer yet neither is sensitive or specific enough for early detection in the general population.

We investigated whether other WAP genes might play a role in ovarian cancer and found that the trio of HE4, SLPI, and Elafin is overexpressed and secreted by HGSOCs [4, 5]. Elafin is an endogenous serine protease inhibitor which, under inflammatory conditions, prevents excessive damage caused by human neutrophil elastase and other serine proteases [6]. It possesses a wide range of activities including antimicrobial properties against viruses, fungi and bacteria, and anti-inflammatory properties that can modulate the immune response [6].

**Figure 1:**
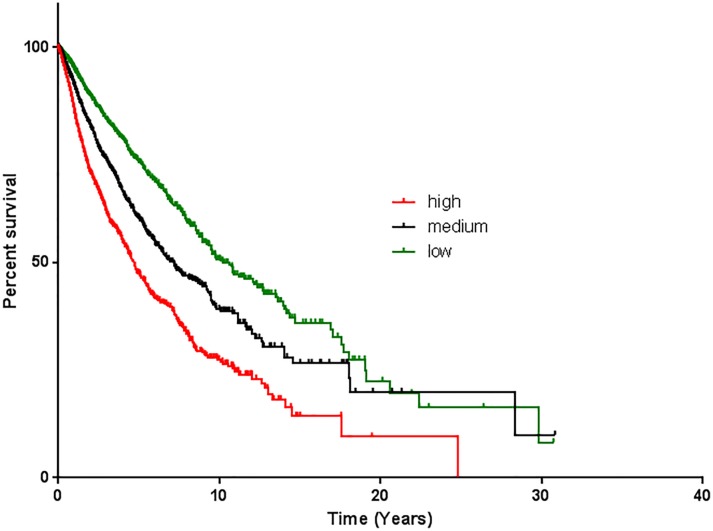
Elafin is associated with poor overall survival Kaplan-Meier overall survival analysis of 9630 patient tumor samples based on the PI3 gene expression data of the TCGA pan-cancer cohort (UCSC Xena Cancer Genomics Browser; https://genome-cancer.soe.ucsc.edu/proj/site/xena/heatmap/) [8]. The tumors were analyzed based on high expression (red line; expression >1.42; n=3145; median survival: 1713 days), medium expression (black line; expression −3.11 to 1.42; n=3095; median survival: 2617days) and low expression (green line; expression < −3.11; n=3390; median survival: 3738 days). Y-axis shows percentage survival and X-axis shows overall survival (OS) in months. P value <0.0001.

Like HE4, Elafin is also implicated in cancer with a number of studies showing high level expression in squamous cell carcinomas, glioblastoma multiforme (GBM), and HGSOC. Its expression in HGSOC was determined by immunohistochemistry and confirmed in a cohort of over 1,000 HGSOC patient samples using gene expression profiling [4, 5]. These studies also demonstrated that high levels of Elafin expression are associated with poor overall survival [4, 5, 7], a finding that appears to be true across tumor types [8] (Figure 1); yet, interestingly, the underlying mechanism remained unclear. Therefore, we conducted a series of *in vitro* studies to further characterize the biology of Elafin and found that Elafin acts as a mitogen to stimulate cell proliferation [5]. This effect was evident in cells engineered to overexpress Elafin as well as in assays using recombinant Elafin protein. Further, mass spectrometry and reverse phase protein arrays demonstrated that this proliferative effect is mediated by activation of the MEK-ERK signaling pathway. RNA interference, MEK pathway inhibition, and antibodies against Elafin could all independently block the effect, validating the requirement of MEK-ERK signaling for induced proliferation.

Given the similarities between HGSOCs and BLBCs we asked whether Elafin might play a similar role in breast cancer. Over 1,100 breast tumor gene expression profiles were analyzed and demonstrated that Elafin is highly expressed by BLBCs compared to luminal or HER2-positive tumors [5]. In addition, consistent with our findings in HGSOC, BLBC cell lines that express Elafin also secrete it. Importantly, clinical data demonstrate that overexpression of Elafin is associated with shorter overall survival (OS) in breast cancer patients, especially in BLBC patients [5]. The association between HGSOC and BLBC includes at least three independent factors: first, among hereditary forms of breast cancer and ovarian cancer, HGSOC and BLBC are the predominant histological subtypes that develop in *BRCA* mutation carriers; second, in comparison with other breast and ovarian cancer subtypes, the majority of HGSOCs and BLBCs harbor *TP53* mutations and exhibit increased genomic instability; third, both tumors are associated with poor outcome. Taken together, these data suggest that Elafin may serve not only as a biomarker but also a therapeutic target in these settings. Moreover, these data exemplify the value of not only defining biomarkers for early detection but also understanding their biological functions. Those biomarkers that serve double duty (like Elafin), that is disease detection and the potential to be a biologically therapeutic target, may define the most significant tools that can be added to the arsenal of anti-cancer therapeutics. As the field continues to identify such biomarkers, define their biology, and characterize them as potential drug targets, our ability to more specifically and potently inhibit tumor growth and metastasis will significantly improve and dramatically impact patient survival.
